# Effects and safety of oral tolvaptan in patients with congestive heart failure: A systematic review and network meta-analysis

**DOI:** 10.1371/journal.pone.0184380

**Published:** 2017-09-12

**Authors:** Mei-Yi Wu, Tzu-Ting Chen, Ying-Chun Chen, Der-Cherng Tarng, Yun-Chun Wu, Hsien-Ho Lin, Yu-Kang Tu

**Affiliations:** 1 Division of Nephrology, Department of Internal Medicine, Shuang Ho Hospital, Taipei Medical University, New Taipei City, Taiwan; 2 Department of Internal Medicine, School of Medicine, College of Medicine, Taipei Medical University, New Taipei City, Taiwan; 3 Graduate Institute of Epidemiology and Preventive Medicine, College of Public Health, National Taiwan University, Taipei, Taiwan; 4 Department of Pharmacy, Taipei Medical University–Shuang Ho Hospital, New Taipei City, Taiwan; 5 Institute of Clinical Medicine, National Yang-Ming University, Taipei, Taiwan; 6 Division of Nephrology, Department of Medicine, Taipei Veterans General Hospital, Taipei, Taiwan; Kurume University School of Medicine, JAPAN

## Abstract

**Aims:**

Several studies reported treatment benefits of tolvaptan in patients with congestive heart failure (CHF). However, the optimal dosage remains unclear. We aimed to compare different dosage of tolvaptan to determine the optimal dosage in terms of the efficacy and safety.

**Methods:**

We searched MEDLINE, PubMed, EMBASE, Cochrane CENTRAL and ClinicalTrials.gov through Aug 31, 2016. Randomized controlled trials (RCTs) comparing tolvaptan of different dosages or to placebo in patients with CHF were included. We used network meta-analysis to look for the optimal dosage in terms of effectiveness and safety. Urine output, body weight change and change in serum sodium were the main outcomes of efficacy. Adverse effects were the secondary outcomes. Quality was assessed by Cochrane risk-of-bias tool.

**Results:**

Twelve RCTs reporting 14 articles with 5793 patients (mean age, 65.7 ± 11.9 years; 73.7% man) were included. Compared with placebo, the tolvaptan 30 mg had similar effects to tolvaptan 45–90 mg in terms of urine output (mean difference [MD] 2.03 liter; 95% confidence interval [CI] 1.3 to 2.71), body weight change (MD -1.12 kg; 95% CI -1.37 to -0.88) and change in serum sodium (MD 3.06 meq/L; 95% CI 2.43 to 3.68). Compared with placebo, tolvaptan of different dosage showed a non-significant higher risk of adverse effects.

**Conclusions:**

These findings suggest that tolvaptan 30 mg and 45 mg may be the optimum dosage for CHF patients, because of its ability to provide favourable clinical results without greater adverse effects. However, tolvaptan is not beneficial for reducing all-cause mortality in CHF patients.

## Introduction

Congestive heart failure (CHF) is a clinical condition with reduced cardiac output and tissue hypo-perfusion, leading to morbidity and mortality. Patients with CHF typically present with shortness of breath, fatigue, legs edema and exercise intolerance, thereby resulting in poor quality of life, frequent admissions, and a shorter life expectancy. Epidemiologic studies indicate that a total of 670,000 new cases of CHF are diagnosed annually and its incidence and prevalence increase with age [1]. Treatment of CHF, aiming at adequate decongestion of the volume overload state, consists of diuretics, beta-blocker, angiotensin converting enzyme (ACE) inhibitors or angiotensin receptor blockers (ARBs) or angiotensin receptor–neprilysin inhibitor (ARNI), digoxin, and aldosterone antagonists. Common adverse effects of pharmacotherapy are abnormal water homeostasis, worsening kidney function, electrolyte disturbances and drug-drug interactions. The resistance to diuretics and the associated morbidities have led to the development of effective and safe treatment strategies that maximize decongestion but minimize the adverse impact on kidney function [2, 3].

Vasopressin receptor antagonists (VRA), or vaptans, have the unique ability to attain an aquaresis, the excretion of electrolyte-free water without accompanying solutes by directly blocking binding of arginine vasopressin (AVP) to its renal receptors. Thus, vasopressin antagonists has been used for the treatment of CHF in the years [4]. There is also a debate over the optimal dosage of vaptans. Several randomized controlled trials (RCTs) evaluated the efficacy and safety of tolvaptan, a selective V2 receptor antagonist in patients with CHF. The results demonstrate symptomatic improvement in patients with decompensated heart failure. According to the American Heart Association (AHA) guideline and European Society of Cardiology (ESC) guideline, VRA may be prescribed to improve serum sodium concentration in hypervolemic hyponatremia states in CHF patients [5, 6]. While the treatment efficacy of tolvaptan with different dosage have been investigated, no single clinical trial has been undertaken to compare the treatment effects of different dosages of tolvaptan simultaneously.

Although previous RCTs and systematic reviews [7–10] reported treatment benefits of tolvaptan on patients with CHF, the optimal dosage remains unclear. This is because traditional meta-analysis can only make pairwise comparisons and is not well suited to compare multiple treatments, such as different dosages of the same drug. The aim of this systematic review was therefore to use network meta-analysis, an emerging new methodology for multiple treatment comparisons, to synthesize all available evidence from RCTs comparing different dosages of tolvaptan in patients with CHF to identify the best treatment strategies.

## Methods

### Literature searches

We undertook electronic literature searches within the MEDLINE, PubMed, EMBASE and Cochrane databases from their inceptions up to Aug 31, 2016. We also searched the World Health Organization International Clinical Trials Registry Platform (http://www.controlled-trials.com) and reference lists of relevant review articles. The following MeSH search headings were used: heart or cardiac or congestive, failure, vasopressin antagonist, tolvaptan. These terms and their combinations were also searched as text-words. The ‘related articles’ facility in PubMed was used to broaden the search, and all retrieved abstracts, studies, and citations were reviewed. The protocol for this systematic review was registered in PROSPERO (CRD42012002061). In addition, we attempted to identify other studies by hand-searching the reference lists of the accessed papers and by contacting known experts in the field. No language restrictions were applied. (S1 Appendix)

### Criteria for study inclusion

We included all published RCTs evaluating the effects of tolvaptan on patients with CHF, either in acute or chronic condition. The full text of potentially relevant studies was carefully reviewed to ensure they satisfied following criteria: (1) the studies prospectively enrolled patients who had confirmed as CHF (2) patients were randomized to receive tolvaptan versus placebo or at least two different dosage of tolvaptan. (3) 1 or more of the primary or secondary outcomes were reported. The studies were excluded if the outcomes of interest were not clearly reported; extraction or calculation of treatment effects from the published results was not possible; or an overlap was present between patient cohorts evaluated by two or more studies.

### Primary and secondary outcomes

We used the following outcomes to evaluate the efficacy and safety of tolvaptan for CHF: (1) body weight loss; (2) urine output; (3) change of serum sodium; (4) mortality; (5) thirsty; (6) renal failure; (7) incidence of all adverse events.

Weight loss was defined as changes in body weight between baseline and follow-up body weights after treatment. Urine volumes were collected on the first day of treatment. Change in serum sodium was measured before and after the first day of treatment. Reported adverse events including mortality, thirsty, renal failure, and incidence of all adverse events were recorded and analyzed. The incidence of renal failure is defined as an increase in serum creatinine (≥0.3 mg/dL increase from baseline) during the period of observation [11, 12].

### Data extraction and risk of bias assessment

Two reviewers (Mei-Yi Wu and Ying-Chun Chen) independently screened all titles and abstracts identified in the literature search, reviewed the full texts of eligible studies, and extracted the following information from each study: name of the first author, year of publication, study population characteristics, study design, inclusion and exclusion criteria, matching criteria, experimental drugs administration, net urine output, weight loss, parameters of the renal function, and complications using a data extraction form. The original authors of the identified studies were contacted for additional information when deemed necessary. Two reviewers independently assessed the risk of bias of each study for each outcome including selection bias, performance bias, detection bias, attrition bias, reporting bias and other bias according to the risk of bias tool developed by the Cochrane Collaboration [13]. Any disagreement between the 2 reviewers was resolved by consulting the senior author (Y-K Tu).

### Data synthesis and analysis

We estimated weighted mean difference (WMD) for weight loss, net urine output, and change in serum sodium, and relative risk (RR) for adverse effects between different treatments. We firstly did pair-wise meta-analyses for direct comparisons using DerSimonian and Laird random-effect model [14]. Statistical heterogeneity was assessed using the I^2^ statistic, with values greater than 50% indicating substantial heterogeneity [15].

Network meta-analysis used both direct and indirect comparisons to estimate relative efficacy of different treatments. We used the network suite of commands written by Ian White implemented in the statistical software package STATA (version 13.0) to undertake network meta-analysis. The probabilities of treatment ranking for each tolvaptan dosage was obtained by simulations, and results were presented by means of rankograms, surface under the cumulative ranking curve (SUCRA), and mean ranks [16]. All the analyses were conducted using STATA (version 13, StataCorp, College Station, Texas, USA). The level of statistical significance was set at 5%, and all statistical tests were 2-sided. For the potential inconsistency within our network meta-analyses, we evaluated the loop-specific inconsistency models, node-splitting models, and the design-by-treatment interaction models [17].

## Results

**Fig 1:**
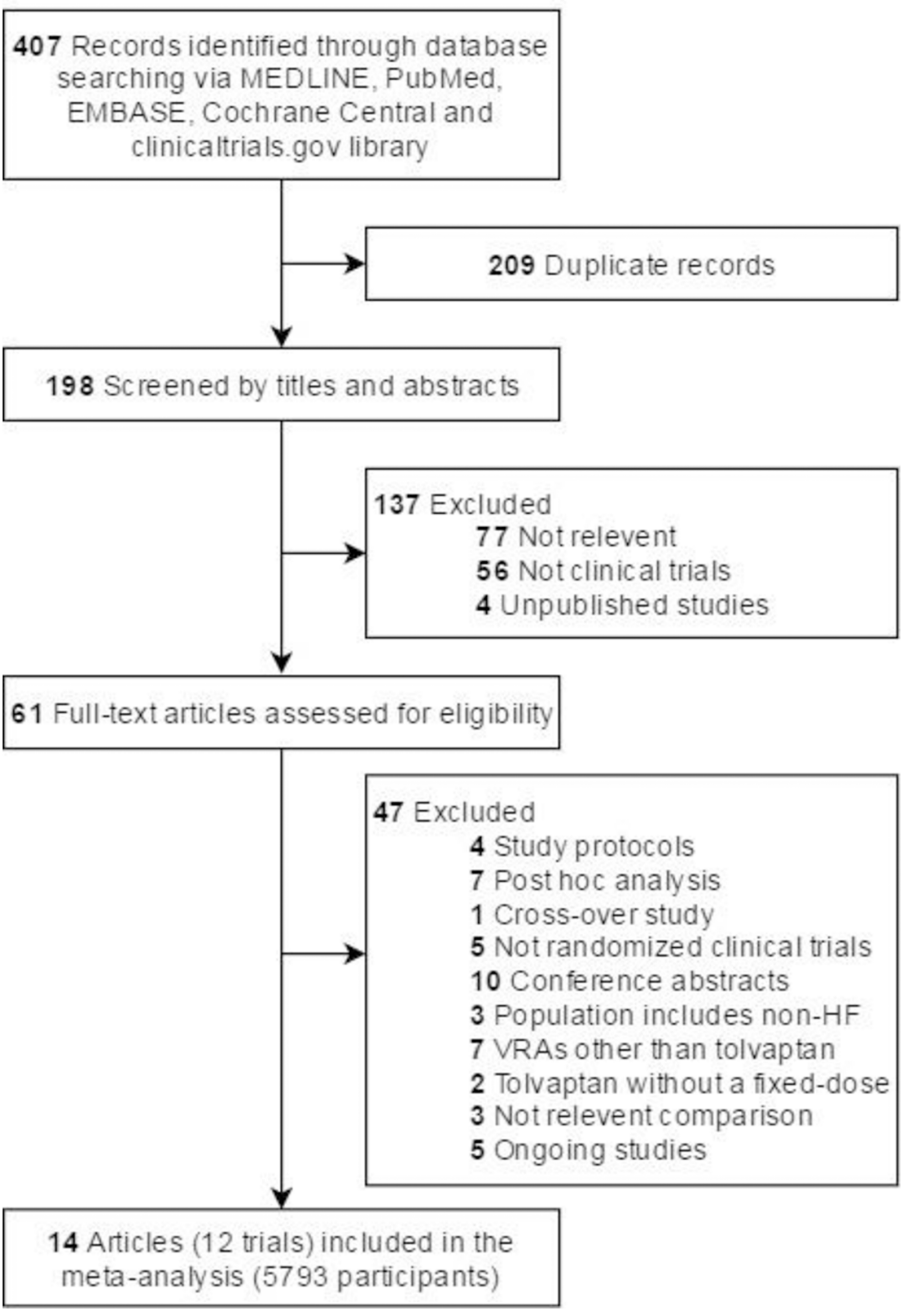
Flow diagram of literature search and trial selection. Abbreviations: HF, heart failure and VRAs, vasopressin receptor antagonists.

Fig 1 showed the flow chart for the electronic searching process. Our initial search strategy yielded 198 citations, 137 of which were excluded based on our screening of titles and abstracts. Full texts of 61 studies were then obtained and reviewed; eventually 14 articles of 12 RCTs were eligible for our systematic review [11, 12, 18–28], and 5793 participants in these 12 RCTs were included in our network meta-analyses.

Characteristics and baseline patient information from each of the 14 articles included in our systematic review were shown in Table 1. The studies were published between 2003 and 2016, and their sample sizes ranged from 20 to 2085. All the trials enrolled patients with CHF who received tolvaptan of various dosages. Mean age of patients in these trials ranged from 57 to 79 years old, and 73.7% of participants were male (in the range of 43–90.5%). NYHA classes of CHF were reported except one. The follow-up length of the included trials ranged from 7 days to more than 1 year. The tolvaptan dosages were adjusted according to various protocols, ranging from 7.5 to 90 mg/d. Durations of tolvaptan therapy ranged from 1 to 365 days. Serum creatinine levels in all CHF patients were below 3 mg/dl. Diuretic therapy was prescribed by 12 studies [26, 28]. Five RCTs [11, 12, 18, 19, 25] were performed exclusively in patients with acute decompensated heart failure (ADHF), whereas 7 RCTs [20, 21, 23, 24, 26–28] were conducted in patients with CHF. Six trials evaluated the urine output after tolvaptan treatment [11, 12, 18, 20, 24, 26]. Eight trials evaluated the body weight loss after tolvaptan treatment [11, 12, 18–21, 23, 26]. Change of serum sodium was reported in 8 trials [11, 12, 18–20, 25, 26, 28]. The safety of tolvaptan was evaluated by the occurrence of mortality, thirsty, renal failure, and adverse events.

**Table 1:** Characteristics of included randomized controlled trials.

Study	Country	Study subject	Intervention of tolvaptan dose(mg)/ duration(days)	No. of patients (% of Male)	Age (y), mean (SD)	NYHA class	LVEF (%), mean (SD)	Serum Cr (mg/dL), mean (SD)	Body weight (kg), mean (SD)	Diuretics	Max follow- up
Gheorghiade et al,[19] 2007 (EVEREST, short-term)	North/South America and Europe	ADHF	[Trial A] C: Placebo E3: 30/ 7	C: 1030 (76.1) E3: 1018 (74.0)	C: 65.6 (11.9) E3: 65.8 (11.7)	Class III or IV	C: 27.3 (8.3) E3: 27.2 (8.2)	C: 1.4 (0.5) E3: 1.3 (0.5)	C: 83.2 (19.0) E3: 82.6 (19.2)	keep	7 days
			[Trial B] C: Placebo E3: 30/ 7	C: 1031 (74.8) E3: 1054 (72.8)	C: 65.6 (12.2) E3: 66.0 (11.7)	Class III or IV	C: 27.7 (8.1) E3: 27.8 (7.7)	C: 1.4 (0.7) E3: 1.4 (0.5)	C: 83.0 (18.2) E3: 84.0 (19.1)		
Gheorghiade et al,[18] 2004 (ACTIV)	the U.S. and Argentina	ADHF	C: Placebo E3: 30/ 60 E5: 60/ 60 E6: 90/ 60	C: 80 (75.0) E3: 78 (68.0) E5: 84 (59.5) E6: 77 (79.2)	C: 60 (14) E3: 62 (14) E5: 62 (13) E6: 62 (14)	C: 93.8%^a^ E3: 91.0% ^a^ E5: 96.5% ^a^ E6: 97.4% ^a^	C: 25 (7) E3: 24 (8) E5: 24 (8) E6: 24 (8)	C: 1.75 (0.27) E3: 2.01 (0.85) E5: 1.82 (0.33) E6: 2.07 (0.85)	C: 83.0 (19.4) E3: 87.2 (24.3) E5: 83.5 (21.5) E6: 83.3 (22.2)	keep	61 days
Gheorghiade et al,[20] 2003	the U.S.	CHF	C: Placebo E3: 30/ 25 E4: 45/ 25 E5: 60/ 25	C: 63 (73.0) E3: 64 (62.5) E4: 64 (60.9) E5: 63 (60.3)	C: 65.1 (12.9) E3: 67.6 (10.9) E4: 65.6 (13.1) E5: 68.5 (13.6)	Class I-IV	C: (36.5%)^b^ E3: (40.6%) ^b^ E4: (32.8%) ^b^ E5: (41.3%) ^b^	C: 1.1 (0.3) E3: 1.3 (0.4) E4: 1.1 (0.3) E5: 1.2 (0.5)	NA	keep	28 days
Inomata et al,[21] 2011	Japan	CHF	E1: 7.5/ 7 E2: 15/ 7	E1: 10 (40) E2: 10 (80)	E1: 69.1 (13.6) E2: 66.8 (10.2)	Class II or III	E1: 49.2 (19.5) E2: 34.1 (16.6)	≦3.0	E1: 61.3 (14.2) E2: 62.7 (13.1)	keep FURO	10 days
Jujo et al,[11] 2016	Japan	ADHF	C: FURO 40 mg IV E1: 7.5/ 5	C: 30 (57) E1: 30 (43)	C: 79 (11) E1: 79 (11)	Class IV	C: 45 (33–55) E1: 46 (37–60) *median (IQR)	C: 1.03 (0.49) E1: 1.01 (0.66)	NA	hold FURO	6 months
Konstam et al,[22] 2007 (EVEREST, long-term)	North/South America and Europe	ADHF	C: Placebo E3: 30/ 60	C: 2061 (75.4) E3: 2072 (73.4)	C: 65.6 (12.0) E3: 65.9 (11.7)	Class III or IV	C: 27.5 (8.2) E3: 27.5 (8.0)	NA	C: 83.1 (18.6) E3: 83.3 (19.1)	keep	9.9 months (median)
Matsue et al,[12] 2016 (AQUAMARINE)	Japan	ADHF	C: Conventional therapy E2: 15/ 2	C: 110 (63.3) E2: 110 (66.7)	C: 72.95 (10.24) E2: 72.99 (8.9)	C: 39.3% ^a^ E2: 37.4% ^a^	C: 46.8 (16.4) E2: 45.4 (18.1)	C: 1.4 (0.5) E2: 1.5 (0.6)	C: 63.6 (17.5) E2: 62.8 (15.4)	keep	90 days
Matsue et al,[29] 2016 (AQUAMARINE, follow-up)	Japan	ADHF	C: Conventional therapy E2: 15/ 2	C: 110 (63.3) E2: 110 (66.7)	C: 72.95 (10.24) E2: 72.99 (8.9)	C: 39.3% ^a^ E2: 37.4% ^a^	C: 46.8 (16.4) E2: 45.4 (18.1)	C: 1.4 (0.5) E2: 1.5 (0.6)	C: 63.6 (17.5) E2: 62.8 (15.4)	keep	636 days (median)
Matsuzaki et al,[23] 2011 (Phase II)	Japan	CHF	C: Placebo E2: 15/ 7 E3: 30/ 7 E4: 45/ 7	C: 30 (60.7) E2: 29 (53.6) E3: 34 (75.8) E4: 29 (53.6)	C: 67.8 (9.6) E2: 66.9 (9.6) E3: 66.4 (12.5) E4: 62.6 (12.5)	Class I-IV	NA	NA	C: 57.7 (12.6) E2: 58.9 (12.8) E3: 60.2 (12.5) E4: 69.8 (16.2)	keep FURO	7 days
Matsuzaki et al,[24] 2011 (QUEST, Phase III)	Japan	CHF	C: Placebo E2: 15/ 7	C: 57 (68.4) E2: 53 (66.0)	C: 71.0 (10.9) E2: 71.3 (10.6)	Class I-IV	C: 50.8 (18.8) E2: 48.3 (20.1)	C: (87.7%)^c^ E2: (81.1%)^c^	C: 56.8 (13.0) E2: 60.9 (13.0)	keep	27 days
Shanmugam et al,[25] 2016	India	ADHF	C: Placebo E2: 15/ 5	C: 26 (65.4) E2: 25 (76.0)	C: 57 (12.0) E2: 58.9 (12.1)	NA	C: 29.2 (8.7) E2: 31.9 (12.2)	C: 1.3 (0.69) E2: 1.3 (0.56)	NA	keep	30 days
Udelson et al,[26] 2011	the U.S.	CHF	C: Placebo E3: 30/ 7 FURO 80mg PO FURO 80mg PO+ 30/ 7	C: 21 (90.5) E3: 20 (75.0) 22 (86.4) 20 (70.0)	C: 58.0 (9.4) E3: 57.3 (9.0) 58.8 (14.2) 60.9 (9.3)	Class II or III	C: 27 (7) E3: 22 (9) 26 (9) 24 (8)	C: 1.0 (0.4) E3: 0.9 (0.2) 1.0 (0.4) 1.0 (0.4)	NA	hold	8 days
Udelson et al,[28] 2008 (ECLIPSE)	the U.S., Romania and Bulgaria	CHF	C: Placebo E2: 15/ 1 E3: 30/ 1 E5: 60/ 1	C: 48 (83.3) E2: 44 (72.7) E3: 43 (83.7) E5: 46 (78.3)	C: 58.9 (14.0) E2: 60.3 (11.7) E3: 59.7 (13.4) E5: 61.0 (11.9)	Class III or IV	C: 24 (9) E2: 23 (8) E3: 23 (9) E5: 24 (7)	< 3.0	NA	hold	8 hrs
Udelson et al,[27] 2007	the U.S.	CHF	C: Placebo E3: 30/ 365	C: 120 (81) E3: 120 (82)	C: 63 (12) E3: 65 (12)	Class II or III	C: 23.7 (5.2) E3: 23.0 (5.0)	C: 1.3 (0.5) E3: 1.3 (0.4)	C: 92.1 (20.9) E3: 85.6 (17.7)	keep	1 year

Abbreviations: ADHF, Acute decompensated heart failure; CHF, Chronic heart failure; C, Control group; E, Experimental group (E1 = 7.5 mg/ E2 = 15 mg/ E3 = 30 mg/ E4 = 45 mg/ E5 = 60 mg/ E6 = 90 mg); FURO, Furosemide; NA, Not available

^a^ NYHA lass III-IV (%)

^b^ LVEF <40% (%)

^c^ Serum Cr <2 mg/dL (%)

**Fig 2:**
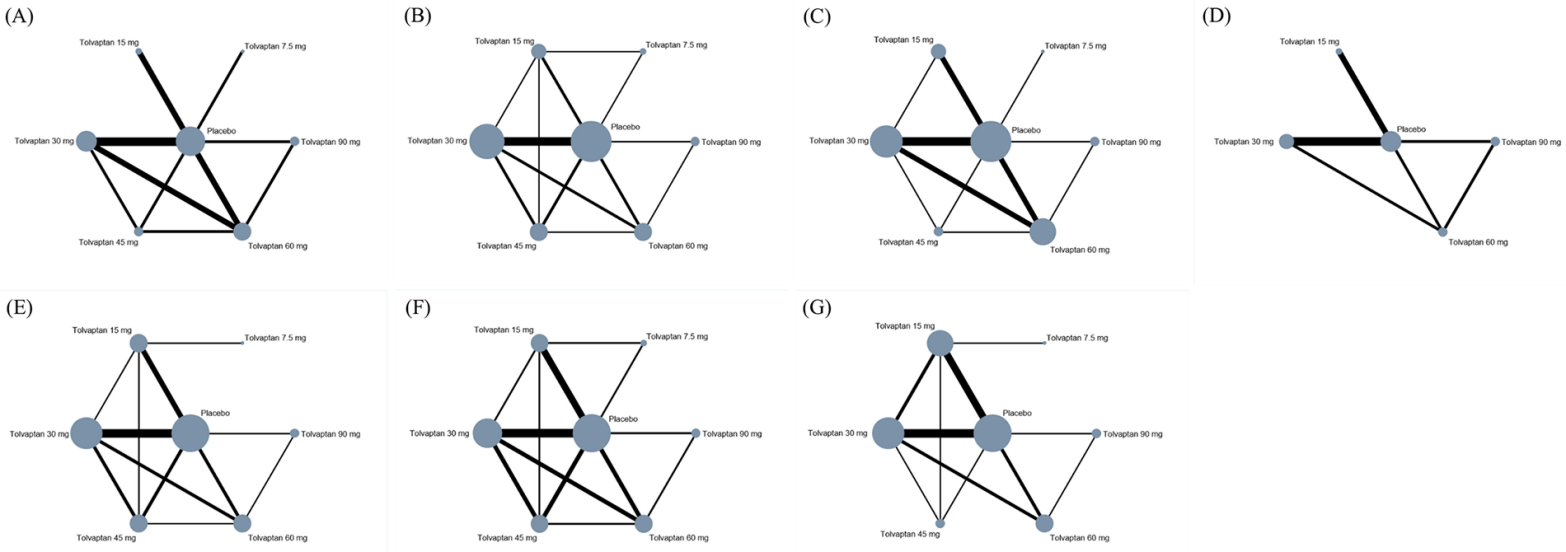
Network geometry for outcomes in network meta-analysis of tolvaptan dosage strategies for CHF. (A) Urine output 994 participants (6 trials). (B) Body weight change 5020 participants (8 trials). (C) Change of serum sodium 4623 participants (8 trials). (D) Mortality 5019 participants (5 trials). (E) Thirst 5223 participants (8 trials). (F) Renal failure 5150 participants (8 trials). (G) Incidence of all adverse effects 5336 participants (8 trials).

Fig 2 showed the network plots for the primary and secondary outcomes. The size of each node is proportional to the number of patients receiving the treatment, and the width of each line is proportional to the number of studies directly comparing the two treatments connected by the line.

**Fig 3:**
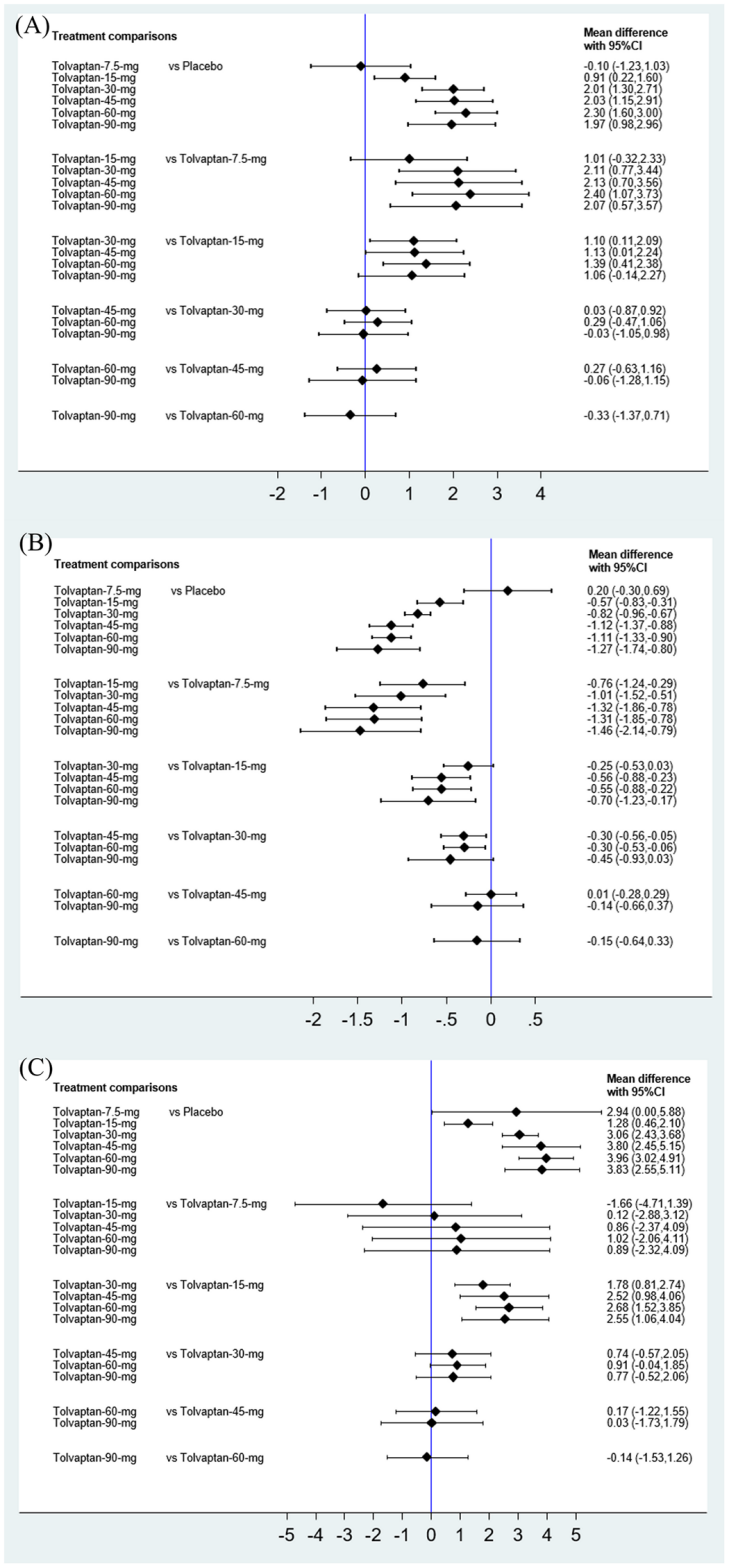
Results from network meta-analysis of primary outcomes. (A) Urine output. (B) Body weight change. (C) Change of serum sodium.

Results from network meta-analysis and meta-analysis of direct comparisons and for primary outcomes were shown in the Fig 3 and S1–S3 Figs, respectively. Results for secondary outcomes such as mortality, thirst, renal failure, and adverse events were shown in S4 and S5 Figs, respectively.

### Primary outcomes—Urine output, body weight change, change in serum sodium

The network meta-analysis for the 3 primary outcomes included 6 to 8 trials involving 994 to 5020 CHF patients (Fig 3). For urine output, no difference was found between tolvaptan 7.5 mg and placebo. Tolvaptan 15 mg or greater dosages yielded on average 0.91 to 2.3 liter more urine output compared to placebo (Fig 3A): tolvaptan 15 mg, 0.91 Liter (L) (95% confidence interval [CI], 0.22–1.60 L); tolvaptan 30 mg, 2.01 L (95% CI, 1.30–2.71 L); tolvaptan 45 mg, 2.03 L (95% CI, 1.15–2.91 L); tolvaptan 60 mg, 2.3L (95% CI, 1.60–3.0 L); and tolvaptan 90 mg, 1.97 L (95% CI, 0.98–2.96 L). Network meta-analysis also showed that tolvaptan 30 mg to 90 mg had similar effects on urine output.

For body weight loss, no difference was found between tolvaptan 7.5 mg and placebo. Tolvaptan 15 mg to 90 mg yielded significantly greater body weight loss than placebo (Fig 3B): tolvaptan 15 mg, -0.57 kg (95% CI, -0.83, -0.31 kg); tolvaptan 30 mg, -0.82 kg (95% CI, -0.96, -0.67 kg); tolvaptan 45 mg, -1.12 kg (95% CI, -1.37, -0.88 kg); tolvaptan 60 mg, -1.11 kg (95% CI, -1.33, -0.9 kg); and tolvaptan 90 mg, -1.27 kg (95% CI, -1.74, -0.8 kg). Network meta-analysis also showed that tolvaptan 45 mg to 90 mg seemed to attain greater body weight loss than smaller doses of tolvaptan.

Tolvaptan yielded greater increases in serum sodium than placebo (Fig 3C): tolvaptan 7.5 mg, 2.94 meq/L (95% CI: 0.0–5.88 meq/L); 15 mg, 1.28 meq/L (95% CI, 0.46–2.1 meq/L); tolvaptan 30 mg, 3.06 meq/L (95% CI, 2.43–3.68 meq/L); tolvaptan 45 mg, 3.80 meq/L (95% CI, 2.45–5.15 meq/L); tolvaptan 60 mg, 3.96 meq/L (95% CI, 3.02–4.91 meq/L); and tolvaptan 90 mg, 3.83 meq/L (95% CI, 2.55–5.11 meq/L). Network meta-analysis also suggested that tolvaptan 30 mg to 90 mg had similar effects on increase of serum sodium. Four trials reported the incidence of hypernatremia, ranging from 0.5–8% in the tolvaptan group [12, 19, 22, 25].

### Secondary outcomes—Mortality, thirsty, renal failure, incidence of all adverse effects

The network meta-analysis for the 4 secondary outcomes included 5 to 8 trials involving 5019 to 5336 CHF patients. In network meta-analysis, there was no significant difference in the risk of the secondary outcome-mortality between interventions. Tolvaptan with dosage 15 mg or above with an increased RR of being thirsty compared with placebo. There was no significant difference in the risk of being thirsty among different dosages of tolvaptan. Details of the comparisons between various dosages of tolvaptan and placebo in mortality, thirst, renal failure, and all incidence of adverse effect are shown in the S5 Fig.

#### Ranking probability

We summarize the rankings of the 7 different dosage strategies with SUCRA probabilities in terms of body weight change, urine output, change of serum sodium, mortality, thirst, renal failure, and adverse effects—with detailed provided in the S6 Fig.

### Risk of bias of included studies

The methodological quality of the 12 included RCTs (14 articles) is assessed by the tool for risk of bias [13]. Overall, studies were considered to be at a low risk of bias. Overall and study-level risks of bias assessments are summarized in S7 Fig in the supplement. Seven studies described whether or how patient allocation to different treatment groups was concealed from the participants (50%). Reporting on blinding was inadequately addressed by most included studies but all were considered at low risk of bias because the outcome of interest were laboratory outcome and unlikely to be influenced by a lack of blinding. All studies were at a low risk of attrition bias. Four studies did not report relevant information so the risk of incomplete data bias was low. Eleven studies were funded by pharmaceutical companies and were judged to be at high risk of bias. Other potential biases included small study group, short follow-up time, and industrial sponsor on authorship. We found no evidence of inconsistency.

## Discussion

Our network meta-analysis of 12 RCTs with 5793 CHF patients showed that CHF patients treated with different dosages of tolvaptan yielded greater body weight loss, increased urine output and greater change in serum sodium compared with placebo. We also found that doses of 30 mg or greater appeared to show similar treatment effects in terms of urine output. Doses of 45 mg or greater appeared to show similar treatment effects on body weight change. Conversely, tolvaptan was associated with higher risk of thirsty compared with placebo, but no significant differences were found between different dosages of tolvaptan. These findings suggest that tolvaptan 30 mg to 45 mg might be considered the optimal dosage for CHF patients, because of its favorable clinical results and similar risks for adverse effects.

In patients with CHF, there is an increased level of AVP, contributing to symptoms such as edema, congestion, and dyspnea. Treatment goals of CHF are to decrease congestion, afterload, and neurohormonal activation in order to improve hemodynamics and symptoms and, perhaps, reduce in-hospital events, re-hospitalizations, and mortality while avoiding toxicities of therapy [30]. Patients with CHF related hyponatremia are associated with increase in mortality [31]. Diuretics is a convenient and low-cost treatment in acute decompensated heart failure. However, the shortcoming of conventional diuretics is the development of resistance and side effects (electrolyte abnormalities, activation of the sympathetic nerve system and the renin-angiotensin-aldosterone system, worsening renal function and ototoxicity) [32]. Tolvaptan, a selective V2 receptor antagonist, reduced cardiac preload resulting in decreased body weight and improved edema and serum sodium without affecting blood pressure or renal function in patients with CHF. It promotes free-water excretion without electrolyte imbalance but has no effect on long-term outcome [19, 20, 22]. In 2009, United States Food and Drug Administration (FDA) approved tolvaptan in the treatment of hypervolemic or normovolemic hyponatremia including patients with heart failure, liver cirrhosis and syndrome of inappropriate anti-diuretic hormone (SIADH) [33]. Hypernatremia was seldom reported in the tolvaptan-treated groups, but it should be closely monitored for safety concern. The principal finding of our network meta-analysis is that tolvaptan treatment has efficacy for CHF patients with few adverse effects and dosage recommendation is 30–45 mg for clinical usage.

Renal insufficiency and CHF are frequently co-existed, which is called organ cross-talk and led to well-known definition of cardiorenal syndrome (CRS). Loop diuretics are frequently used for CKD patients to ameliorate congestion in those with CRS. The studies included in the network meta-analysis were conducted in subjects with near-normal kidney function or mildly impaired kidney function (serum creatinine < 3 mg/dl). Future study should focus on whether tolvaptan is also safe and effective on urine volume and serum sodium excretions in CHF patients with late stage CKD.

Although adverse events were observed more frequently in the tolvaptan-treated groups than in the placebo group, our results indicated that the incidences of renal failure and all adverse effects and mortality were similar. Only the incidence of thirsty was significantly higher in tolvaptan treatment group compared with placebo group.

Although null effects in morbidity and mortality were found in our network meta-analysis, some studies showed potentially benefit in certain populations. There have been few studies conducted to investigate the tolvaptan effect on mortality in CHF patients [18, 22, 24, 27, 29]. In a post hoc analysis study, total mortality was lower in the tolvaptan groups compared with placebo in patients with elevated BUN levels and severe systemic congestion at randomization [18]. Because this is a post hoc analysis, these conclusions need to be interpreted cautiously due to high drop-out rate. Matsue et al. also reported short-term, early tolvaptan treatment in ADHF with renal dysfunction showed a neutral effect on prognosis but potential benefit among the patients with relatively preserved renal function [29]. The long-term beneficial effect of tolvaptan on mortality should be validated in large-scale, randomized controlled trials with a longitudinal follow-up. No benefit of tolvaptan on reducing all-cause mortality in CHF patients was found in the network meta-analysis.

It is unclear in the previous literature about optimal dosage of tolvaptan in terms of the safety and adverse effects. Matsuzaki et al. reported that tolvaptan at doses of 15–45 mg/day exhibited dose-dependency in urine volume, but not in body weight change [23]. Our network meta-analysis compared tolvaptan of different dosages in the same model and found that 30mg of tolvaptan improved urine output, whereas increasing dosage adds little urine output but possible increase in adverse effects. Our results suggest that tolvaptan 45 mg increased body weight loss, whereas increasing dosage provides little further benefit. This network meta-analysis is the first to assess efficacy, safety and optimal dosage of vasopressin antagonist tolvaptan. Our results show that increasing dosage beyond tolvaptan 45 mg provides little additional benefit to body weight change and urine output, and therefore future trials with dosage above 45 mg are probably unwarranted. Taking into account the cost-effectiveness, tolvaptan 30 mg is recommended.

Compared to previous meta-analyses, our network meta-analysis has several strengths: first, we considered relevant outcomes (that is, urine output, weight loss, change of serum sodium, thirsty, renal failure, incidence of all adverse effects and all-cause mortality). Also, we used a comprehensive search in multiple databases and all languages and are unlikely to have missed important numbers of relevant paper. Finally, we did a network meta-analysis to evaluate the optimal dosage. Rather than grouping various dosages into one treatment group, our network meta-analysis evaluated the efficacy of different dosages separately within a single statistical model. Network meta-analysis also allowed us to compare different dosages indirectly when no head-to-head trial was available. Our findings need to be considered as average effects because we did not have individual patient data which would allow us to identify potential differential effects of available dosage of tolvaptan in subgroup of CHF patients.

Our network meta-analysis has also several limitations: First, detailed dosages of diuretic and inotropic agents were not available. Diuretic may cause electrolyte abnormalities, such as hypokalemia and hyponatremia. Second, population characteristics, tolvaptan dosage, renal function status, concurrent medications in included studies would contribute to the observed heterogenicity. The applied analysis is based on relative treatment effect and the analysis accounts for these differences by preserving randomization. Third, the length of follow-up varied across studies, resulting in potential variations in event rates in the adverse effects. Our analysis does not incorporate costs, future studies need to be conducted to determine the treatment period and the most cost-effective management option.

## Conclusions

In summary, patients treated with tolvaptan showed greater weight loss and urine output than placebo group. The adverse effects were similar between the two groups except for thirsty. Our study has several implications for clinical practice. Clinicians can consider more widespread use of tolvaptan for the patients with CHF which might result in more appropriate treatment and symptom relief. From a clinical perspective, tolvaptan is a promising decongestive treatment to achieve symptomatic improvement and favorable clinical outcomes. Taking all evidence into account, tolvaptan 30-45mg is the most effective dosage.

## Supporting information

S1 Fig Forest plot of direct comparison: Tolvaptan versus Control. Efficacy outcome: body weight change at 24-hr.
(PDF)

S2 Fig Forest plot of direct comparison: Tolvaptan versus Control. Efficacy outcome: urine output at 24-hr.
(PDF)

S3 Fig Forest plot of direct comparison: Tolvaptan versus Control. Efficacy outcome: change of serum sodium at 24-hr.
(PDF)

S4 Fig Forest plot of direct comparison: Tolvaptan versus Control. Main secondary outcomes: 4.1.1 Thirst/ 4.1.2 Renal failure/ 4.1.3 Incidence of all adverse effects/ 4.1.4 Mortality.
(PDF)

S5 Fig Main secondary outcomes of thirst, renal failure, incidence of all adverse effects and mortality. (A) Mortality(B) Thirst(C) Renal failure(D) Incidence of all adverse effects
(PDF)

S6 Fig Ranking Probability of Strategies and Surface under the Cumulative Curve in the Network Meta-analysis of tolvaptan dosage strategies for CHF patients. (A) Urine output(B) Body weight change(C) Change of serum sodium(D) Mortality(E) Thirsty(F) Renal failure(G) Incidence of all adverse effects
(PDF)

S7 Fig **(A) Risk of bias graph**: Review authors’ judgements about each risk of bias item presented as percentages across all included studies **(B) Risk of bias summary**: Review authors’ judgements about each risk of bias item for each included study.
(PDF)

S1 Appendix Literature search strategy details. (PDF)

S1 Checklist (PDF)
