# Correction: Effects and safety of oral tolvaptan in patients with congestive heart failure: A systematic review and network meta-analysis

**DOI:** 10.1371/journal.pone.0215624

**Published:** 2019-04-11

**Authors:** Mei-Yi Wu, Tzu-Ting Chen, Ying-Chun Chen, Der-Cherng Tarng, Yun-Chun Wu, Hsien-Ho Lin, Yu-Kang Tu

There is an error in affiliation 2 for author Mei-Yi Wu. The correct affiliation 2 is: Division of Nephrology, Department of Internal Medicine, School of Medicine, College of Medicine, Taipei Medical University, Taipei, Taiwan.
