# Supplementary material for: Effects and safety of oral tolvaptan in patients with congestive heart failure: A systematic review and network meta-analysis
Source: PLoS One. 2017 Sep 12;12(9):e0184380. doi: 10.1371/journal.pone.0184380 (PMC5595312; doi:10.1371/journal.pone.0184380)
Supplement: S1 Fig — Efficacy outcome: body weight change at 24-hr. (PDF) [file pone.0184380.s001.pdf]

Supporting Information (S1 Fig)

	Tolvaptan			Control				Mean Difference
Study or Subgroup	Mean	SD	Total	Mean	SD	Total	Weight	IV, Random, 95% CI
Gheorghiade 2003	-0.86	0.79	187	0.32	0.46	62	14.8%	-1.18 [-1.34, -1.02]
Gheorghiade 2004 [ACTIV]	-1.99	1.94	239	-0.6	1.19	80	11.4%	-1.39 [-1.75, -1.03]
Gheorghiade 2007 [EVEREST] (A)	-1.71	1.8	978	-0.99	1.83	997	14.8%	-0.72 [-0.88, -0.56]
Gheorghiade 2007 [EVEREST] (B)	-1.82	2.01	1021	-0.95	1.85	1002	14.7%	-0.87 [-1.04, -0.70]
Jujo 2016 (1)	-1.63	1.58	30	-2.14	1.16	30	6.3%	0.51 [-0.19, 1.21]
Matsue 2016	-1.58	1.33	110	-0.995	1.085	110	12.1%	-0.59 [-0.91, -0.26]
Matsuzaki 2011 [phase II]	-0.93	0.65	89	-0.2	0.47	28	13.9%	-0.73 [-0.95, -0.51]
Udelson 2011	-0.69	0.675	20	-0.155	0.35	21	11.9%	-0.53 [-0.87, -0.20]
**Total (95% CI)**			**2674**			**2330**	**100.0%**	**-0.77 [-1.00, -0.55]**

Heterogeneity: Tau² = 0.08; Chi² = 49.03, df = 7 (P < 0.00001); I² = 86%
Test for overall effect: Z = 6.70 (P < 0.00001)

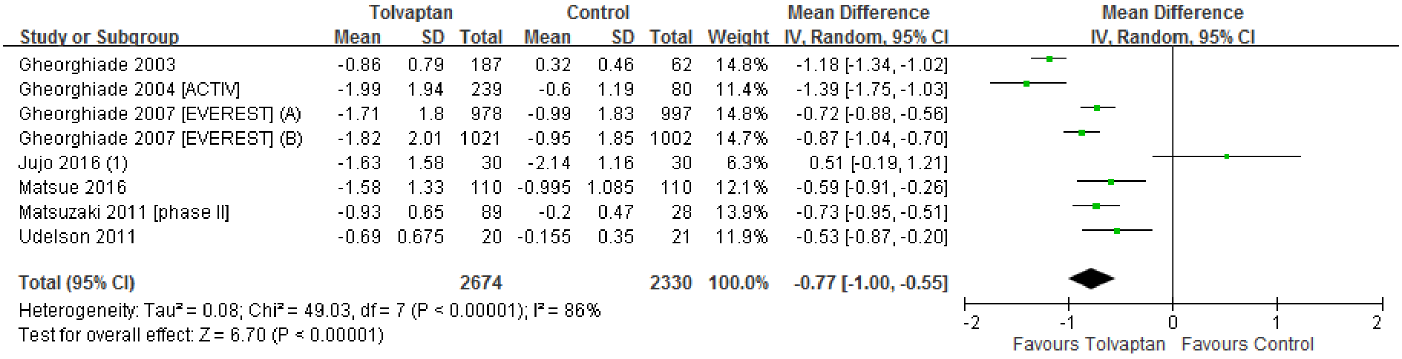

Footnotes
(1) Tolvaptan group: hold furosemide; Control group: Furosemide 40 mg IV

S1 Fig. Forest plot of direct comparison: Tolvaptan versus Control. Efficacy outcome: body weight change at 24-hr
