# Supplementary material for: Effects and safety of oral tolvaptan in patients with congestive heart failure: A systematic review and network meta-analysis
Source: PLoS One. 2017 Sep 12;12(9):e0184380. doi: 10.1371/journal.pone.0184380 (PMC5595312; doi:10.1371/journal.pone.0184380)
Supplement: S2 Fig — Efficacy outcome: urine output at 24-hr. (PDF) [file pone.0184380.s002.pdf]

Supporting Information (S2 Fig)

Study or Subgroup	Tolvaptan Mean	Tolvaptan SD	Tolvaptan Total	Control Mean	Control SD	Control Total	Weight	Mean Difference IV, Random, 95% CI
Gheorghiade 2003	4.22	0.72	187	2.3	0.2	62	18.9%	1.92 [1.81, 2.03]
Gheorghiade 2004 [ACTIV]	4.14	2.38	239	2.3	1.13	80	17.6%	1.84 [1.45, 2.23]
Jujo 2016 (1)	2.11	1.73	30	2.17	0.71	30	15.4%	-0.06 [-0.73, 0.61]
Matsue 2016	3.2	1.6	110	2.5	1.1	110	17.8%	0.70 [0.34, 1.06]
Matsuzaki 2011 [QUEST, phase III]	2.7	0.89	53	1.6	0.54	57	18.3%	1.10 [0.82, 1.38]
Udelson 2011	5.7	2.2	19	2.4	0.8	20	12.0%	3.30 [2.25, 4.35]
**Total (95% CI)**			**638**			**359**	**100.0%**	**1.40 [0.80, 2.00]**

Heterogeneity: Tau² = 0.49; Chi² = 97.28, df = 5 (P < 0.00001); I² = 95%

Test for overall effect: Z = 4.60 (P < 0.00001)

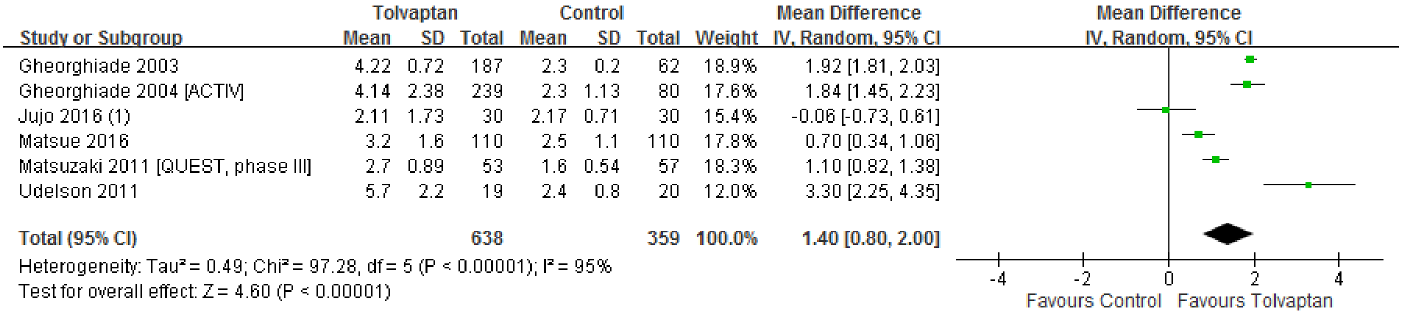

Footnotes

(1) Tolvaptan group: hold furosemide; Control group: Furosemide 40 mg IV

S2 Fig. Forest plot of direct comparison: Tolvaptan versus Control. Efficacy outcome: urine output at 24-hr
