# Supplementary material for: Effects and safety of oral tolvaptan in patients with congestive heart failure: A systematic review and network meta-analysis
Source: PLoS One. 2017 Sep 12;12(9):e0184380. doi: 10.1371/journal.pone.0184380 (PMC5595312; doi:10.1371/journal.pone.0184380)
Supplement: S3 Fig — Efficacy outcome: change of serum sodium at 24-hr. (PDF) [file pone.0184380.s003.pdf]

Supporting Information (S3 Fig)

Study or Subgroup	Tolvaptan Mean	Tolvaptan SD	Tolvaptan Total	Control Mean	Control SD	Control Total	Weight	Mean Difference IV, Random, 95% CI
Gheorghiade 2003	2.66	3.23	187	0	5.2	62	12.1%	2.66 [1.29, 4.03]
Gheorghiade 2004 [ACTIV]	3.22	4	239	-0.2	3.12	80	14.1%	3.42 [2.57, 4.27]
Gheorghiade 2007	3.28	4.11	1743	-0.41	3.53	1772	15.6%	3.69 [3.44, 3.94]
Jujo 2016	4.07	6.05	30	1.13	4.72	30	7.1%	2.94 [0.19, 5.69]
Matsue 2016	1.16	1.76	108	-0.06	1.56	109	15.3%	1.22 [0.78, 1.66]
Shanmugam 2016	2.75	5.21	25	1.15	3.29	26	8.1%	1.60 [-0.80, 4.00]
Udelson 2008 [ECLIPSE]	3.05	4.13	133	-0.7	3.2	48	13.0%	3.75 [2.60, 4.90]
Udelson 2011	2.04	1.12	20	-0.15	1.07	21	14.7%	2.19 [1.52, 2.86]
**Total (95% CI)**			**2485**			**2148**	**100.0%**	**2.71 [1.73, 3.70]**

Heterogeneity: $Tau^2 = 1.60$; $Chi^2 = 100.25$, df = 7 (P < 0.00001); $I^2 = 93\%$
Test for overall effect: Z = 5.41 (P < 0.00001)

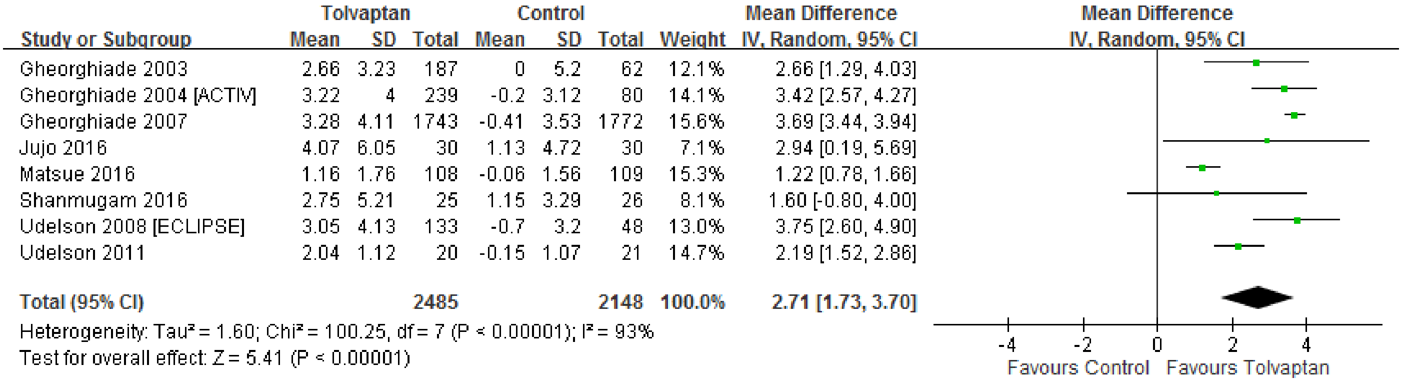

S3 Fig. Forest plot of direct comparison: Tolvaptan versus Control. Efficacy outcome: change of serum sodium at 24-hr
