# Supplementary material for: Effects and safety of oral tolvaptan in patients with congestive heart failure: A systematic review and network meta-analysis
Source: PLoS One. 2017 Sep 12;12(9):e0184380. doi: 10.1371/journal.pone.0184380 (PMC5595312; doi:10.1371/journal.pone.0184380)
Supplement: S4 Fig — Main secondary outcomes: 4.1.1 Thirst/ 4.1.2 Renal failure/ 4.1.3 Incidence of all adverse effects/ 4.1.4 Mortality. (PDF) [file pone.0184380.s004.pdf]

Supporting Information (S4 Fig)

Study or Subgroup	Tolvaptan Events	Tolvaptan Total	Control Events	Control Total	Weight	Risk Ratio M-H, Random, 95% CI
**4.1.1 Thirst**						
Gheorghiade 2003	60	187	3	62	12.8%	6.63 [2.16, 20.39]
Gheorghiade 2004 [ACTIV]	22	238	1	79	5.6%	7.30 [1.00, 53.30]
Konstam 2007	331	2063	43	2055	29.7%	7.67 [5.61, 10.47]
Matsuzaki 2011 [phase II]	30	89	3	29	13.0%	3.26 [1.07, 9.89]
Matsuzaki 2011 [QUEST, phase III]	9	53	1	57	5.4%	9.68 [1.27, 73.84]
Shanmugam 2016	7	25	5	26	14.6%	1.46 [0.53, 3.99]
Udelson 2007	32	120	7	120	18.9%	4.57 [2.10, 9.95]
**Subtotal (95% CI)**		**2775**		**2428**	**100.0%**	**4.84 [2.90, 8.08]**
Total events	491		63			
Heterogeneity: Tau² = 0.20; Chi² = 12.01, df = 6 (P = 0.06); I² = 50%						
Test for overall effect: Z = 6.02 (P < 0.00001)						
**4.1.2 Renal failure**						
Gheorghiade 2003	4	187	0	62	1.0%	3.02 [0.16, 55.24]
Gheorghiade 2004 [ACTIV]	5	238	0	79	1.0%	3.68 [0.21, 65.85]
Jujo 2016	2	30	10	30	4.1%	0.20 [0.05, 0.84]
Konstam 2007	133	2063	140	2055	61.5%	0.95 [0.75, 1.19]
Matsue 2016	26	108	30	109	29.8%	0.87 [0.56, 1.38]
Matsuzaki 2011 [phase II]	3	89	0	29	1.0%	2.33 [0.12, 43.88]
Shanmugam 2016	2	25	1	26	1.6%	2.08 [0.20, 21.52]
**Subtotal (95% CI)**		**2740**		**2390**	**100.0%**	**0.91 [0.68, 1.22]**
Total events	175		181			
Heterogeneity: Tau² = 0.02; Chi² = 6.85, df = 6 (P = 0.33); I² = 12%						
Test for overall effect: Z = 0.63 (P = 0.53)						
**4.1.3 Incidence of all adverse effects**						
Gheorghiade 2004 [ACTIV]	210	238	64	79	19.3%	1.09 [0.97, 1.22]
Konstam 2007	1844	2027	1776	2061	34.1%	1.06 [1.03, 1.08]
Matsue 2016	10	108	6	105	0.6%	1.62 [0.61, 4.30]
Matsuzaki 2011 [phase II]	79	89	18	29	5.7%	1.43 [1.07, 1.92]
Matsuzaki 2011 [QUEST, phase III]	38	53	42	57	8.4%	0.97 [0.77, 1.22]
Udelson 2007	112	120	116	120	29.1%	0.97 [0.91, 1.02]
Udelson 2008 [ECLIPSE]	64	133	16	48	2.8%	1.44 [0.93, 2.24]
**Subtotal (95% CI)**		**2768**		**2499**	**100.0%**	**1.06 [0.98, 1.14]**
Total events	2357		2038			
Heterogeneity: Tau² = 0.00; Chi² = 17.74, df = 6 (P = 0.007); I² = 66%						
Test for overall effect: Z = 1.43 (P = 0.15)						
**4.1.4 Mortality**						
Gheorghiade 2004 [ACTIV]	13	239	7	80	1.3%	0.62 [0.26, 1.50]
Konstam 2007	537	2072	543	2061	93.1%	0.98 [0.89, 1.09]
Matsue 2016, follow-up	26	108	27	109	4.5%	0.97 [0.61, 1.55]
Matsuzaki 2011 [QUEST, phase III]	1	53	2	57	0.2%	0.54 [0.05, 5.76]
Udelson 2007	6	120	11	120	1.1%	0.55 [0.21, 1.43]
**Subtotal (95% CI)**		**2592**		**2427**	**100.0%**	**0.97 [0.88, 1.07]**
Total events	583		590			
Heterogeneity: Tau² = 0.00; Chi² = 2.66, df = 4 (P = 0.62); I² = 0%						
Test for overall effect: Z = 0.59 (P = 0.55)						

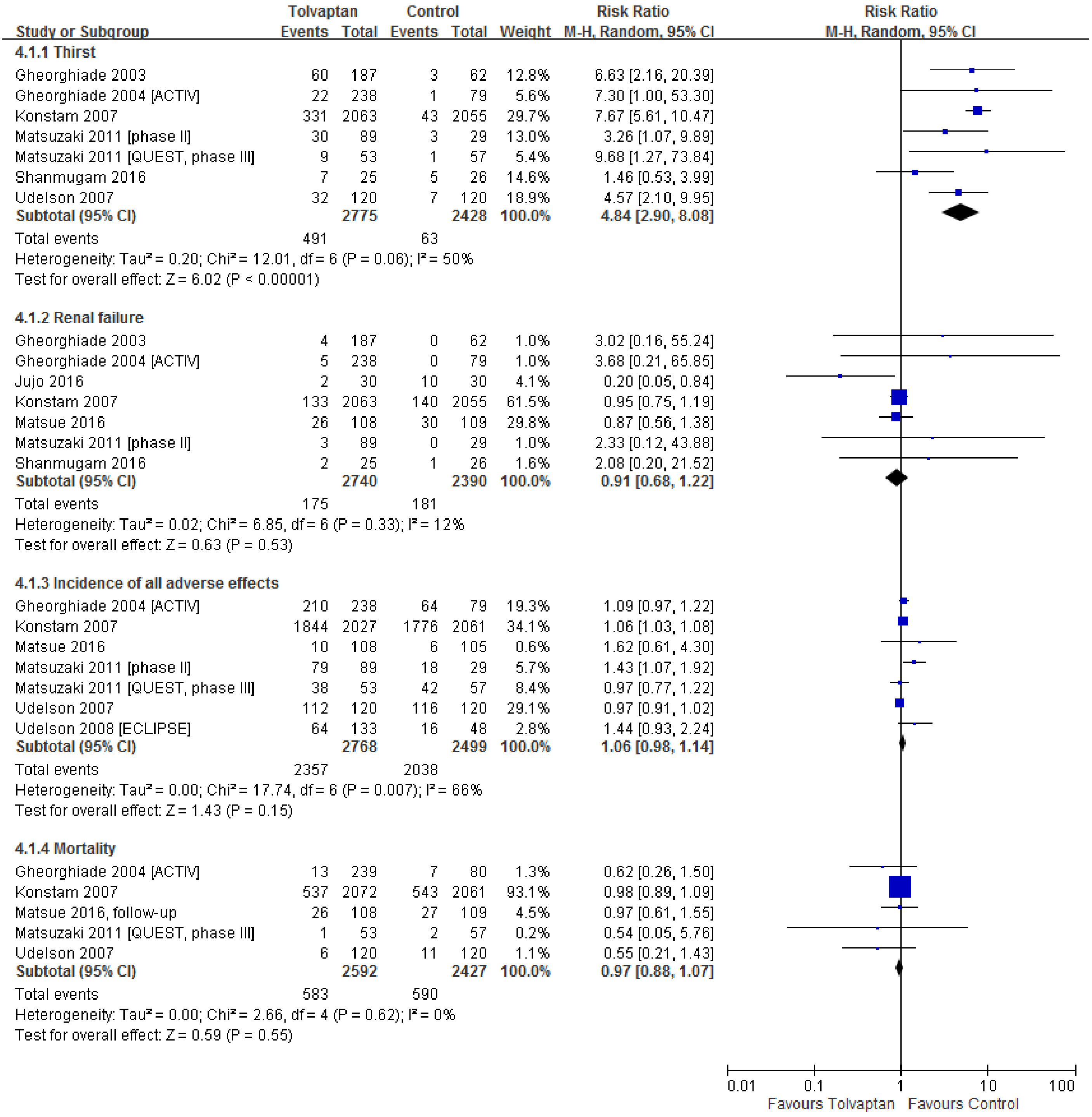

S4 Fig. Forest plot of direct comparison: Tolvaptan versus Control. Main secondary outcomes: 4.1.1 Thirst/ 4.1.2 Renal failure/ 4.1.3 Incidence of all adverse effects/ 4.1.4 Mortality
