# Supplementary material for: Effects and safety of oral tolvaptan in patients with congestive heart failure: A systematic review and network meta-analysis
Source: PLoS One. 2017 Sep 12;12(9):e0184380. doi: 10.1371/journal.pone.0184380 (PMC5595312; doi:10.1371/journal.pone.0184380)
Supplement: S5 Fig — (A) Mortality(B) Thirst(C) Renal failure(D) Incidence of all adverse effects (PDF) [file pone.0184380.s005.pdf]

Supporting Information (S5 Fig)

(A) Mortality

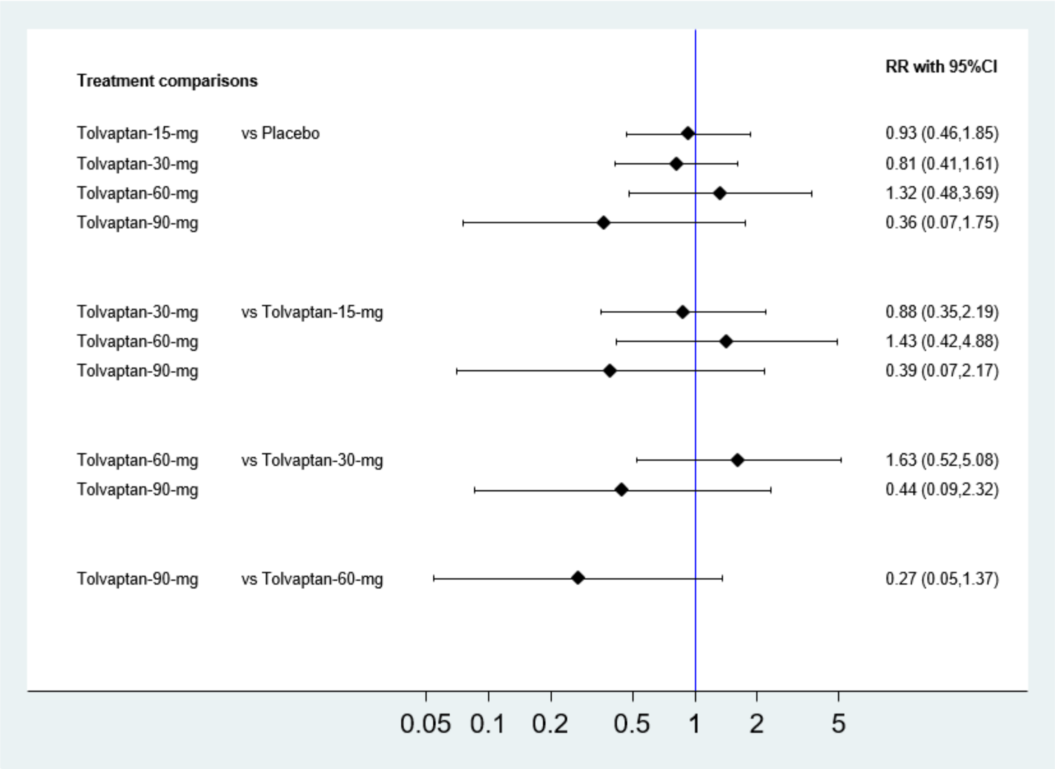

(B) Thirst

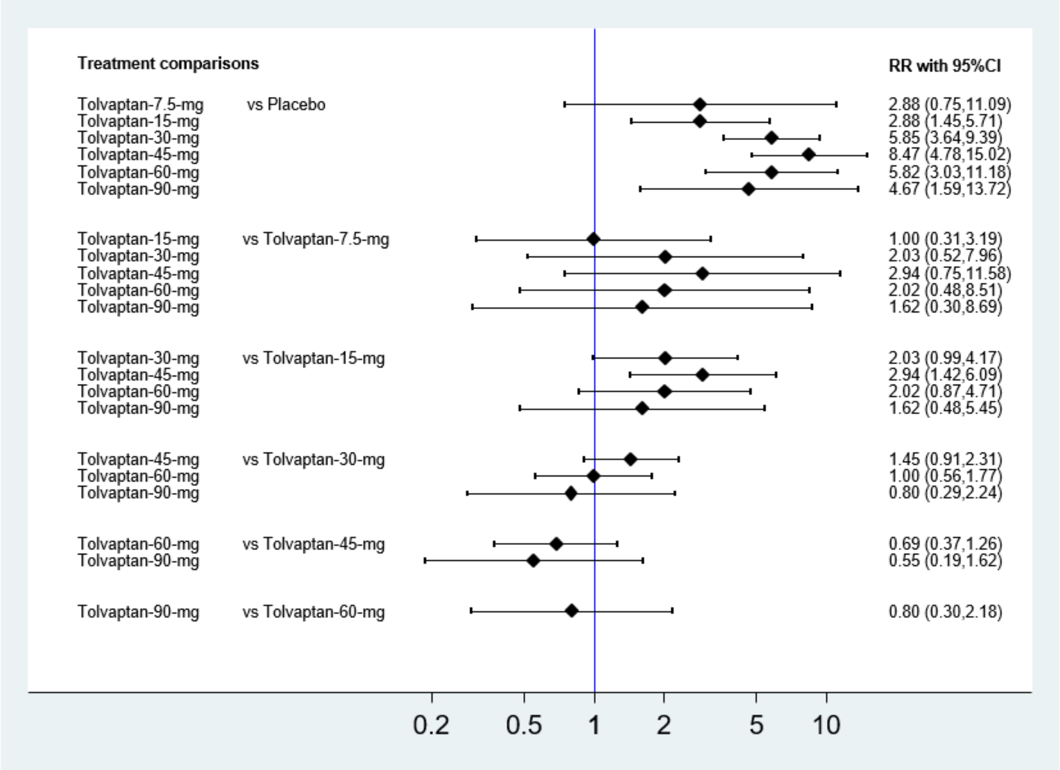

(C) Renal failure

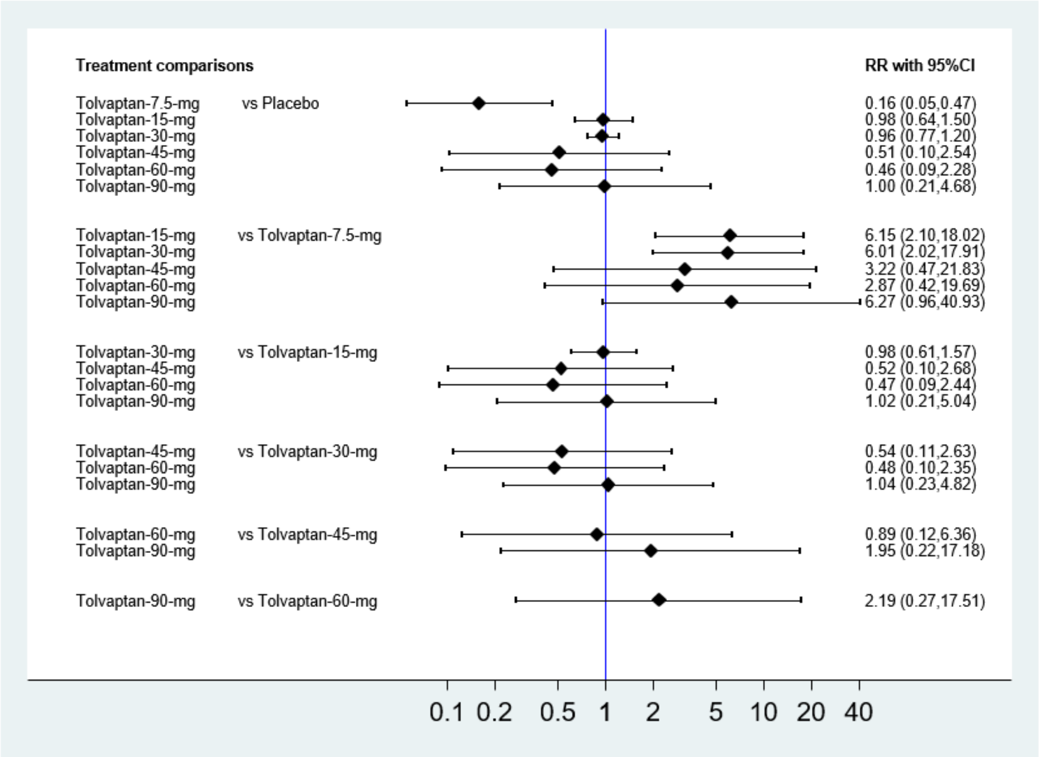

(D) Incidence of all adverse effects

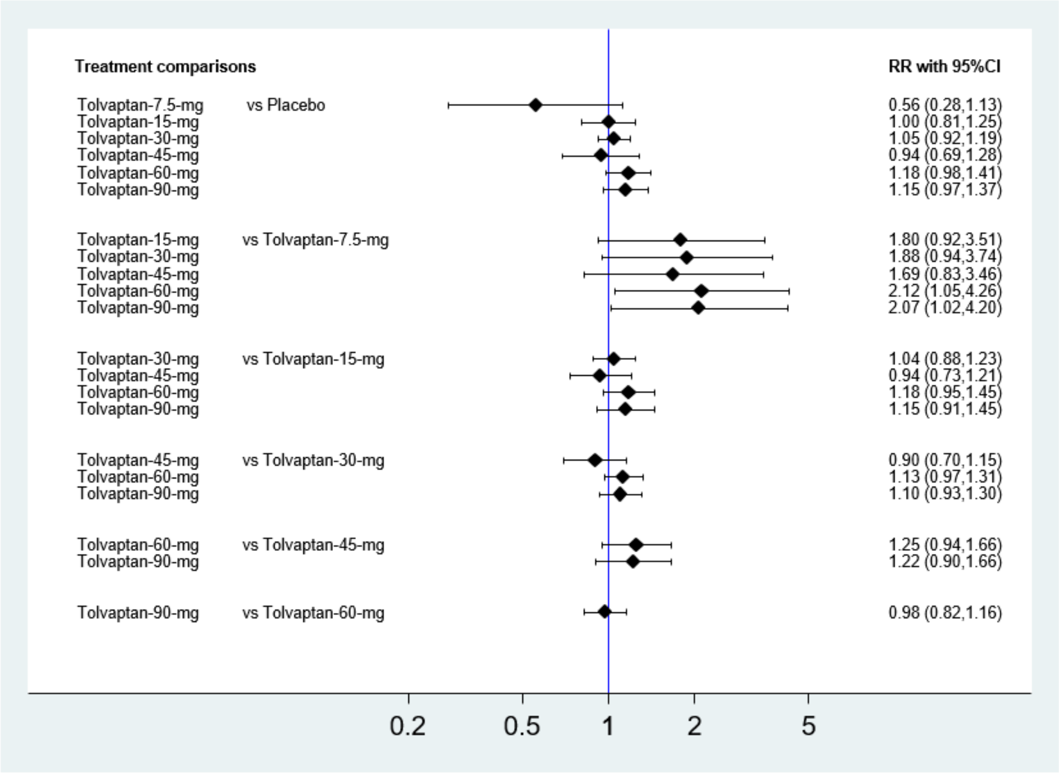

S5 Fig. Main secondary outcomes of thirst, renal failure, incidence of all adverse effects and mortality
