# Supplementary material for: Effects and safety of oral tolvaptan in patients with congestive heart failure: A systematic review and network meta-analysis
Source: PLoS One. 2017 Sep 12;12(9):e0184380. doi: 10.1371/journal.pone.0184380 (PMC5595312; doi:10.1371/journal.pone.0184380)
Supplement: S6 Fig — (A) Urine output(B) Body weight change(C) Change of serum sodium(D) Mortality(E) Thirsty(F) Renal failure(G) Incidence of all adverse effects (PDF) [file pone.0184380.s006.pdf]

Supporting Information (S6 Fig)

(A) Urine output

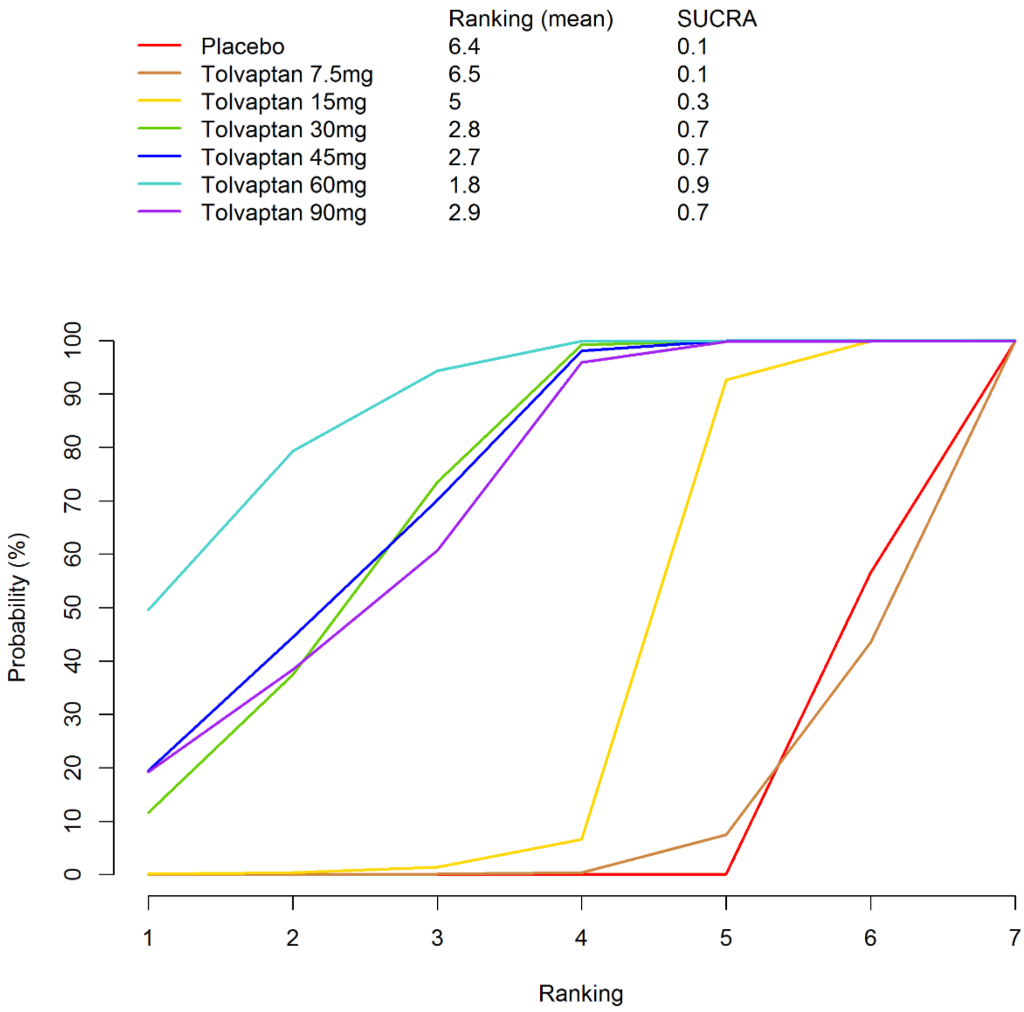

(B) Body weight change

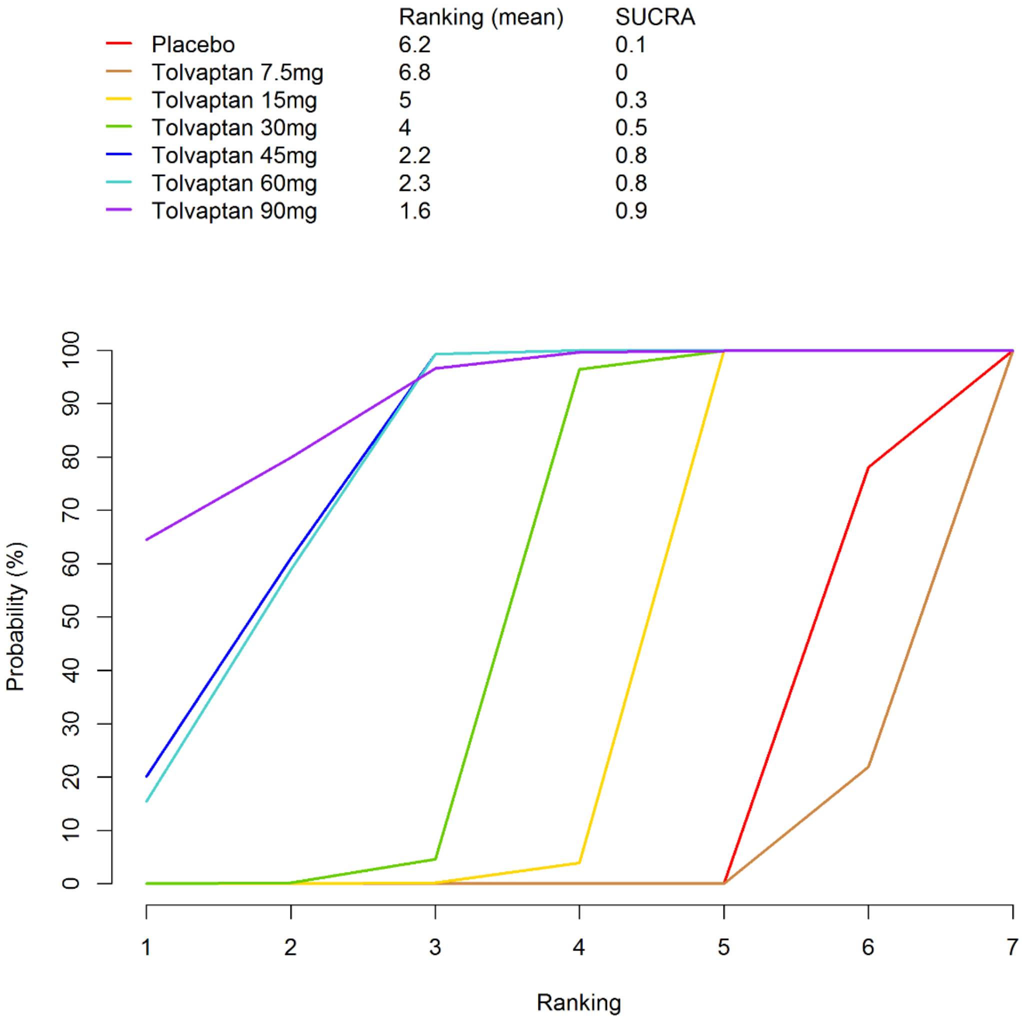

(C) Change of serum sodium

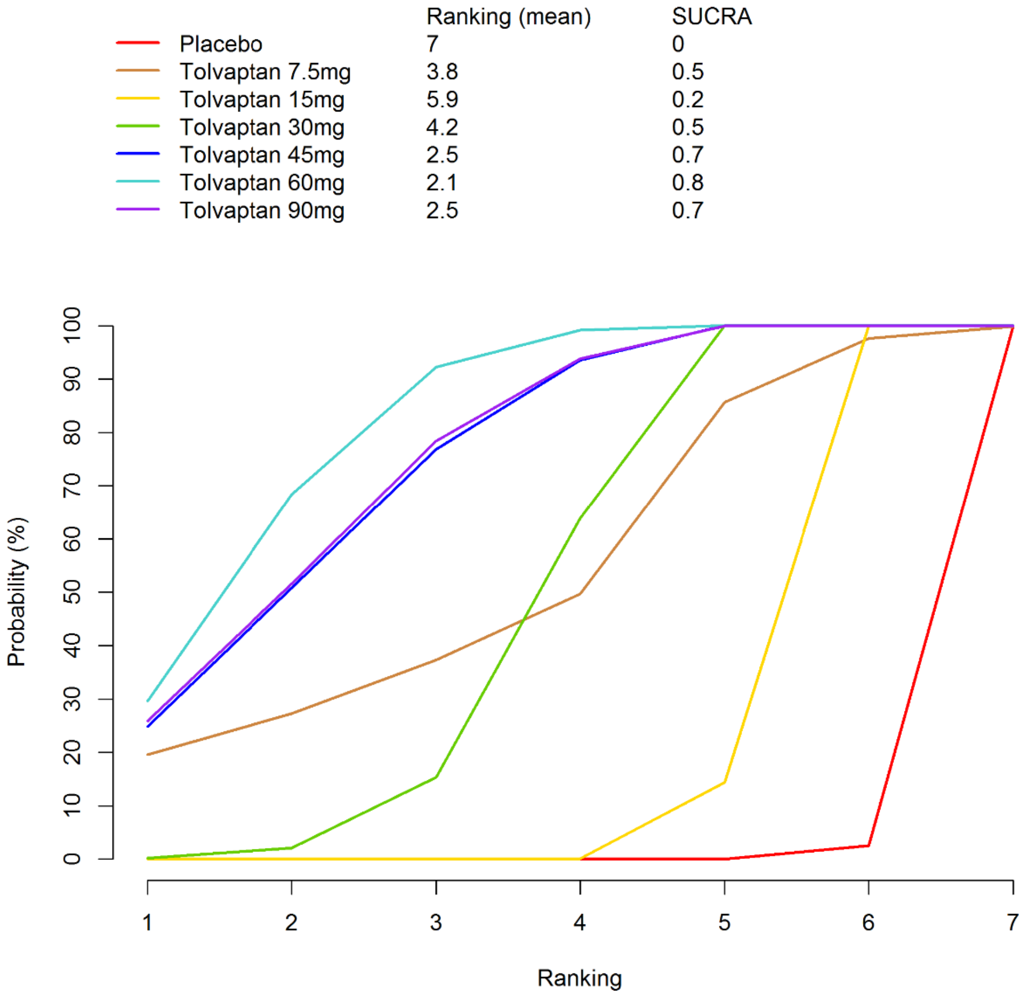

(D) Mortality

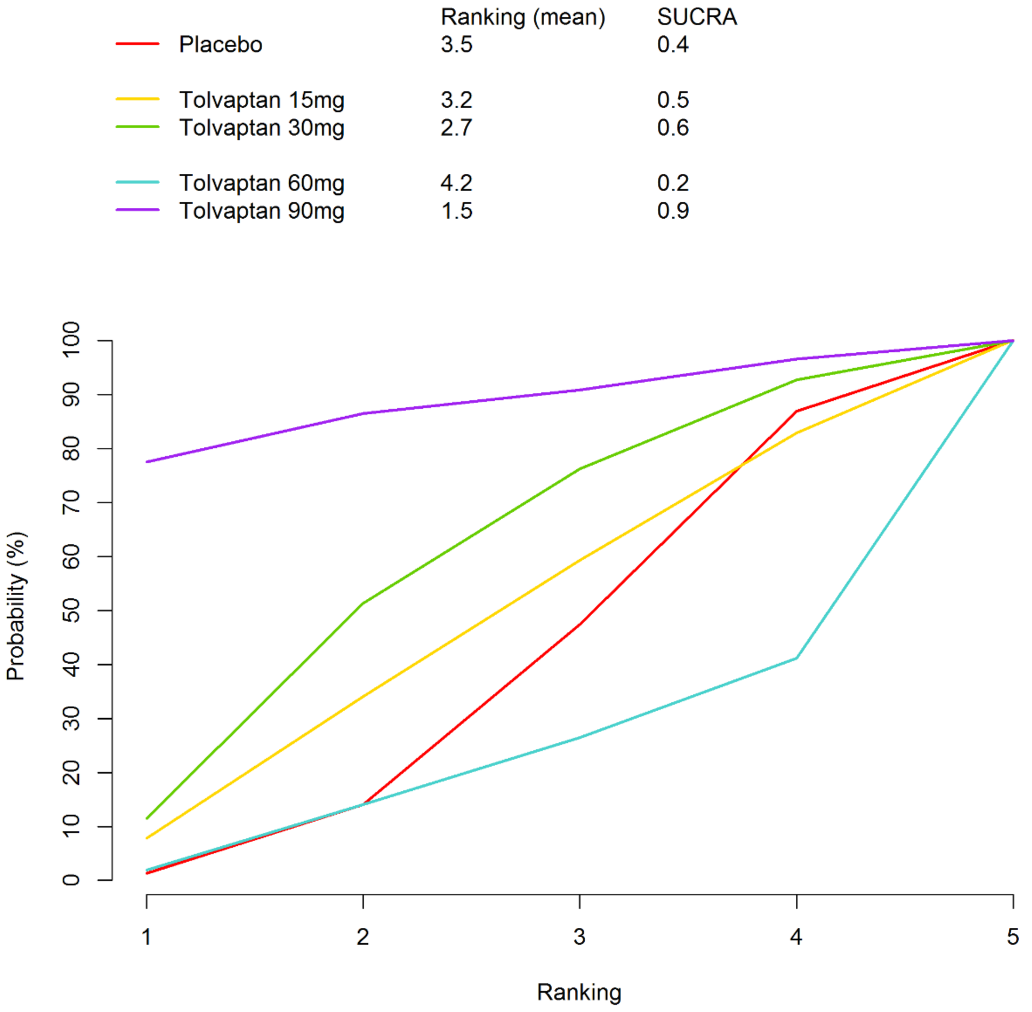
Ranking (mean)
SUCRA
Placebo 3.5 0.4
Tolvaptan 15mg 3.2 0.5
Tolvaptan 30mg 2.7 0.6
Tolvaptan 60mg 4.2 0.2
Tolvaptan 90mg 1.5 0.9
Probability (%)
0
10
20
30
40
50
60
70
80
90
100
1
2
3
4
5
Ranking

(E) Thirsty

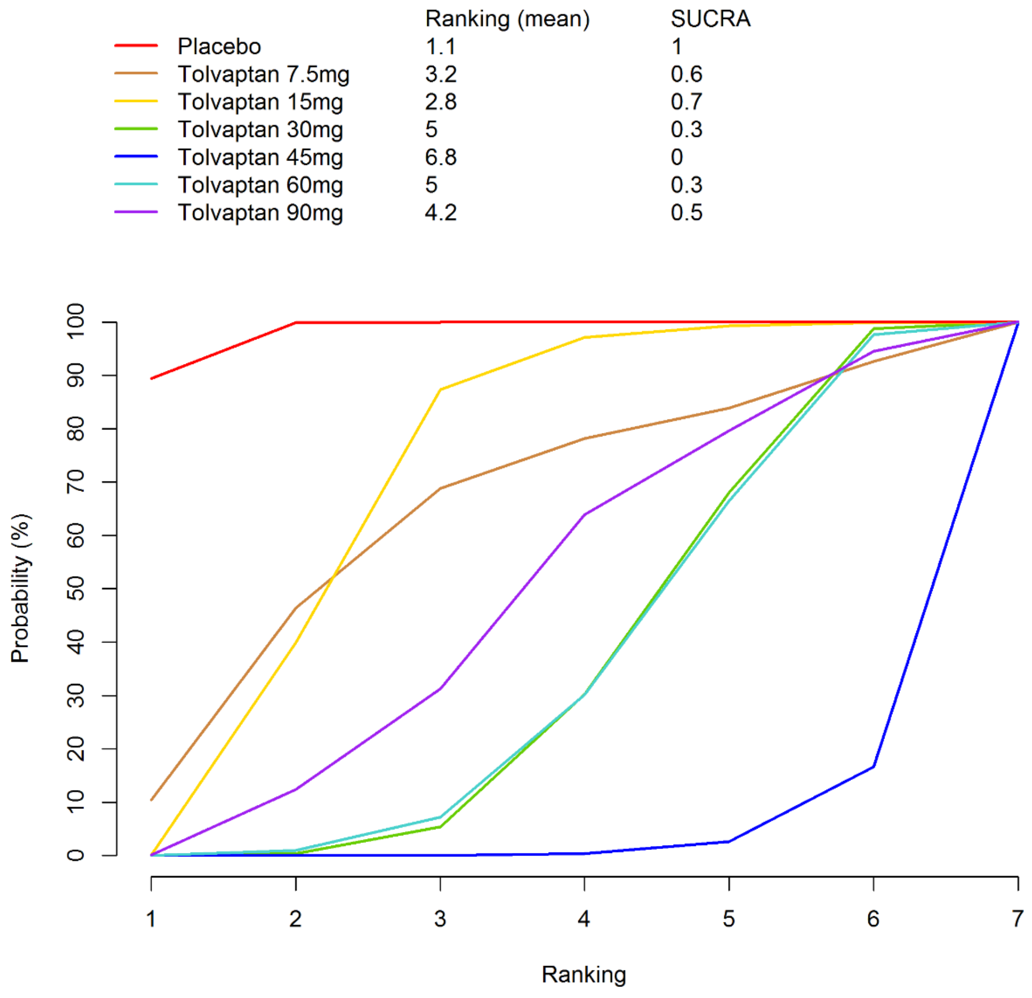
Ranking (mean)
SUCRA
Placebo 1.1 1
Tolvaptan 7.5mg 3.2 0.6
Tolvaptan 15mg 2.8 0.7
Tolvaptan 30mg 5 0.3
Tolvaptan 45mg 6.8 0
Tolvaptan 60mg 5 0.3
Tolvaptan 90mg 4.2 0.5
Probability (%)
0
10
20
30
40
50
60
70
80
90
100
1
2
3
4
5
6
7
Ranking

(F) Renal failure

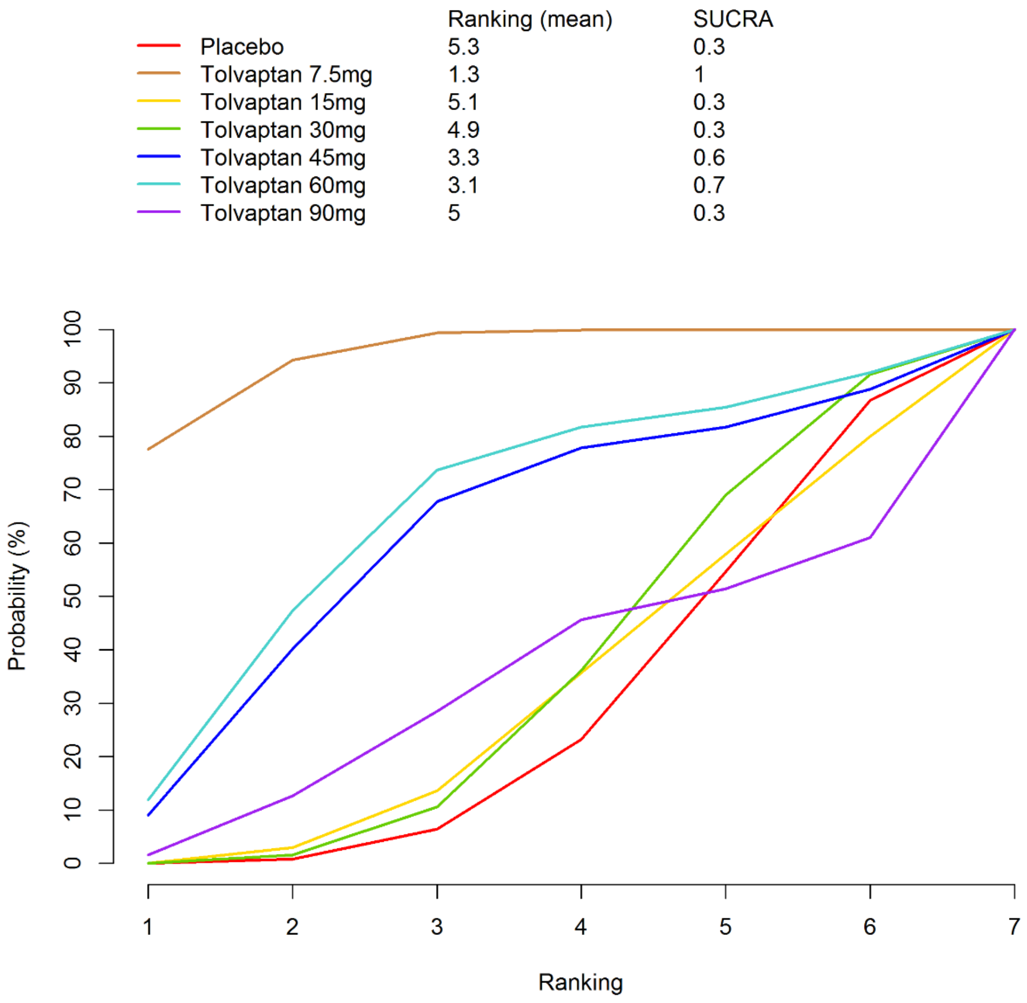

(G) Incidence of all adverse effects

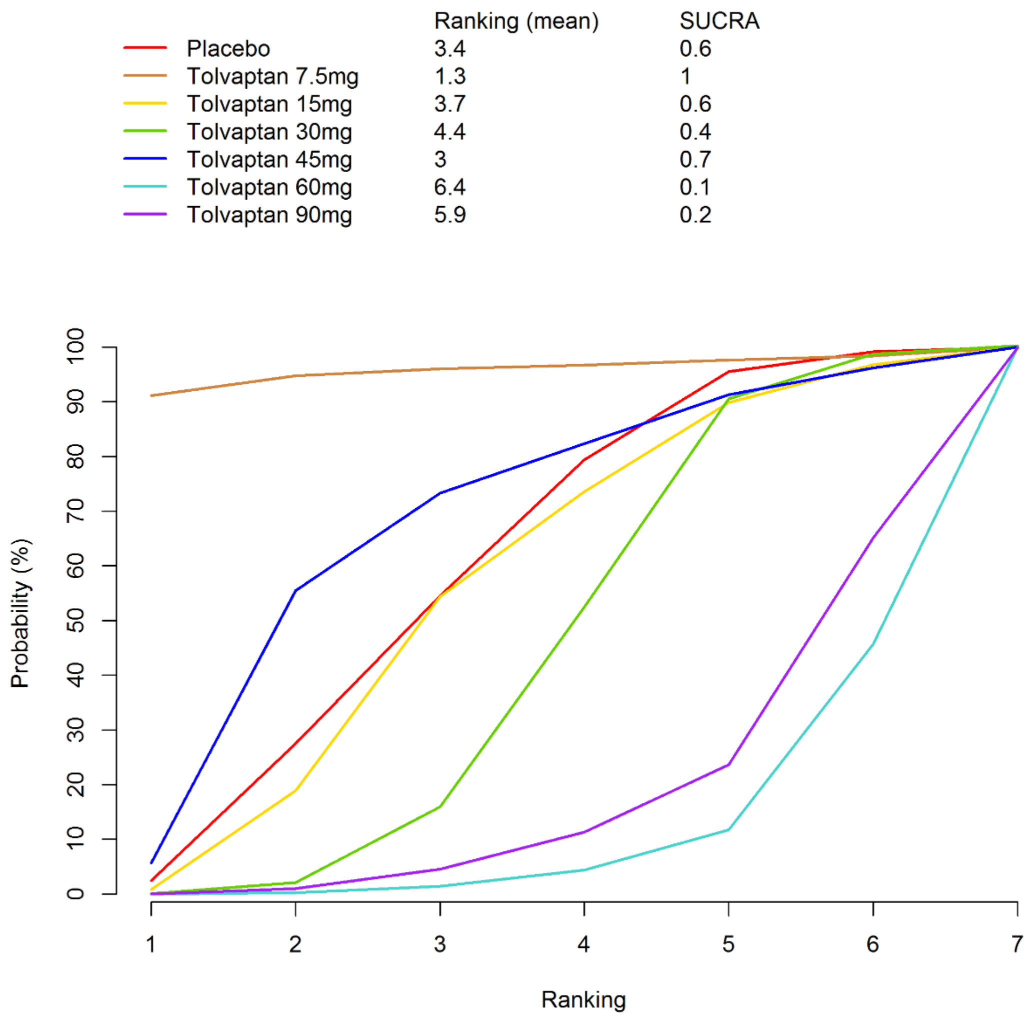

S6 Fig. Ranking Probability of Strategies and Surface under the Cumulative Curve in the Network Meta-analysis of tolvaptan dosage strategies for CHF patients
