# Supplementary material for: Effects and safety of oral tolvaptan in patients with congestive heart failure: A systematic review and network meta-analysis
Source: PLoS One. 2017 Sep 12;12(9):e0184380. doi: 10.1371/journal.pone.0184380 (PMC5595312; doi:10.1371/journal.pone.0184380)
Supplement: S7 Fig — (A) Risk of bias graph: Review authors’ judgements about each risk of bias item presented as percentages across all included studies (B) Risk of bias summary: Review authors’ judgements about each risk of bias item for each included study. (PDF) [file pone.0184380.s007.pdf]

Supporting Information (S7 Fig)

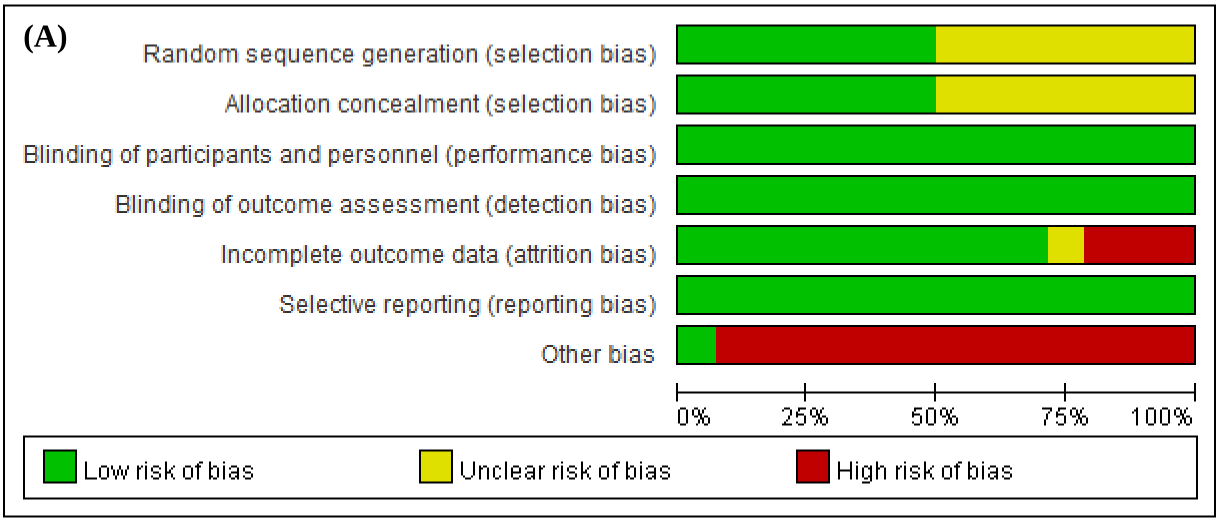

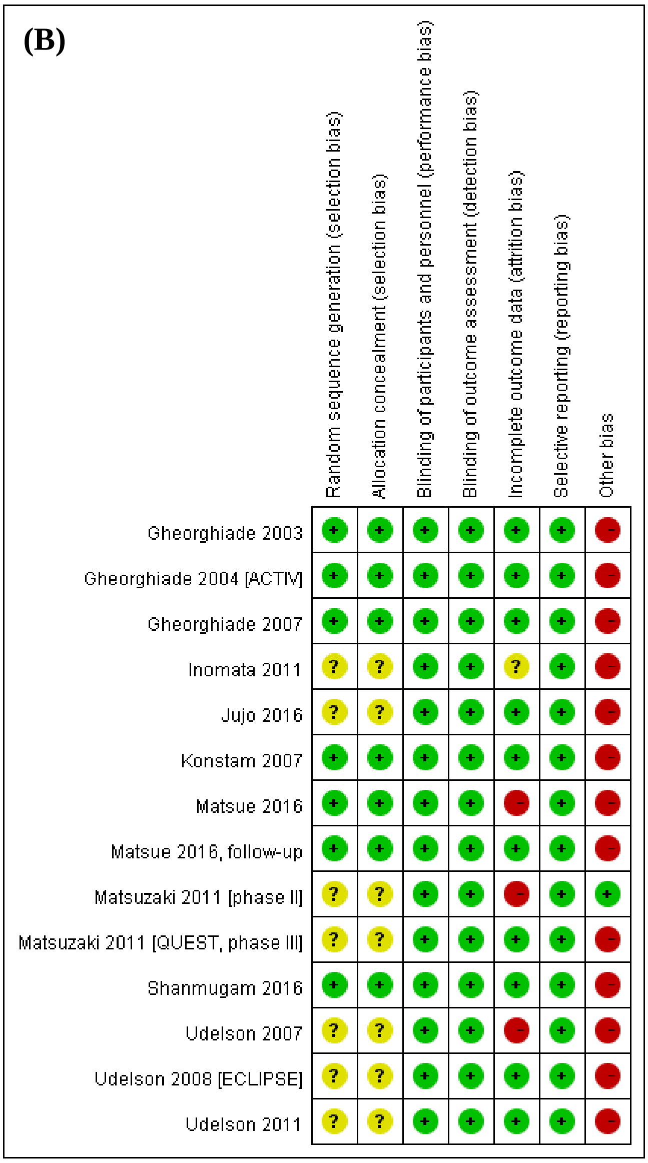

S7 Fig. (A) Risk of bias graph: Review authors' judgements about each risk of bias item presented as percentages across all included studies **(B) Risk of bias summary:** Review authors' judgements about each risk of bias item for each included study
