# Supplementary material for: Effects and safety of oral tolvaptan in patients with congestive heart failure: A systematic review and network meta-analysis
Source: PLoS One. 2017 Sep 12;12(9):e0184380. doi: 10.1371/journal.pone.0184380 (PMC5595312; doi:10.1371/journal.pone.0184380)
Supplement: S1 Appendix — (PDF) [file pone.0184380.s008.pdf]

S9 Appendix: Literature Search Strategy Details

Pubmed search strategy

1. (((heart OR cardiac OR congestive)) AND failure)
2. (vasopressin antagonist* OR vasopressin receptor antagonist* OR tolvaptan OR *vaptan*)
3. Clinical Trial[ptyp] OR Controlled Clinical Trial[ptyp] OR Randomized Controlled Trial[ptyp] OR Clinical Study[ptyp]
4. #1 AND #2 AND #3

EMBASE search strategy

1. 'heart'/exp
2. heart/
3. cardiac/
4. congestive/
5. or/1-4
6. 'failure'/exp
7. 'vasopressin receptor antagonist*'/exp
8. 'vasopressin antagonist*'/exp
9. 'tolvaptan'/exp
10. tolvaptan/
11. vra/
12. v2ra/
13. 'OPC-41061'/
14. or/7-13
15. and/5-6, 14
16. [controlled clinical trial]/lim
17. and/15-16

MEDLINE search strategy

1. ((heart or cardiac or congestive) and failure).mp. [mp=title, abstract, original title, name of substance word, subject heading word, keyword heading word, protocol supplementary concept word, rare disease supplementary concept word, unique identifier]
2. (tolvaptan or 'vasopressin receptor antagonist*' or 'vasopressin antagonist*' or 'v2ra' or 'vra' or 'OPC-41061').mp. [mp=title, abstract, original title, name of substance word, subject heading word, keyword heading word, protocol supplementary concept word, rare disease supplementary concept word, unique

identifier]

3. ('clinical study'/de OR 'clinical trial'/de OR 'clinical trial (topic)'/de OR 'controlled clinical trial (topic)'/de OR 'controlled study'/de OR 'major clinical study'/de OR 'phase 2 clinical trial (topic)'/de OR 'phase 3 clinical trial (topic)'/de OR 'randomized controlled trial'/de OR 'randomized controlled trial (topic)'/de)
4. and/1-3

Cochrane CENTRAL search strategy

1. MeSH descriptor: [Heart Failure] explode all trees
2. heart or cardiac or congestive:ti,ab,kw
3. "failure":ti,ab,kw
4. "tolvaptan" or "vasopressin antagonist" or "vasopressin receptor antagonist" or "*vaptan*":ti,ab,kw
5. [18 and #2 and #3 and #4]

ClinicalTrial.com search strategy

Vasopressin receptor antagonist and Heart failure
